# Effect of the Grafting Reaction of Aluminum Nitride on the Multi-Walled Carbon Nanotubes on the Thermal Properties of the Poly(phenylene sulfide) Composites

**DOI:** 10.3390/polym9090452

**Published:** 2017-09-15

**Authors:** Myounguk Kim, Sunmin Park, Jongshin Park

**Affiliations:** 1 Department of Biosystems & Biomaterials Science and Engineering, Seoul National University, Seoul 08826, Korea; myounguk@snu.ac.kr; 2 Ceramic Fiber & Composite Center, Korea Institute of Ceramic Engineering and Technology, Jinju 52851, Korea

**Keywords:** poly(phenylene sulfide), multi-walled carbon nanotubes, aluminum nitride, grafting reaction, thermal property

## Abstract

In this study, the PPS/MWCNTs/AlN composite was prepared with poly(phenylene sulfide) (PPS), covalent functionalized multi-walled carbon nanotubes (fMWCNTs), and aluminum nitride (AlN) via melt-blending techniques. The AlN is a fascinating non-oxidizing ceramic material having the highest thermal conductivity among the ceramic materials. In order to introduce the functional groups on the surface of the AlN particles, a silane coupling agent was used as it is able to graft with the functional groups on the covalent functionalized MWCNTs. The silanization reaction of the AlN was confirmed qualitatively and quantitatively by FT-IR (Fourier Transform Infrared Spectroscopy), and XPS (X-ray Photoelectron Spectroscopy). The grafting reaction of the AlN particles on the MWCNTs was confirmed using UV–Vis (Ultraviolet-Visible Spectroscopy), FE-SEM (Field-Emission Scanning Electron Microscopy) and FE-TEM (Field-Emission Transmission Electron Microscopy) images. The grafting reaction was accomplished by observing the change of the transmittance, the morphology of the AlN particle bonded to the MWCNTs. For the morphological changes of the fractured surface of the PPS/MWCNTs/AlN composites by FE-SEM, the hybrid filler was homogeneously dispersed on the PPS matrix when the AlN particle was grafted on the MWCNTs. The homogeneous distribution of the hybrid filler acts as a heat transfer path, which led the higher thermal properties, such as thermal conductivity, thermal resistance, and melting temperature than those of not grafted MWCNTs.

## 1. Introduction

Recently, the uses of the super-engineering plastic have steadily increased for various industrial applications due to their higher heat and chemical resistance and physical properties than the commodity polymers have been used. In this study, poly(phenylene sulfide) (PPS), which is one of the super-engineering plastics, was used as a thermal conductive polymer matrix of the composite. The PPS is known for a semi-crystalline aromatic thermoplastic polymer possessing a high melting point ($T_{m}$), at approximately 285 °C, excellent mechanical properties, thermal resistance, and excellent flame and chemical resistance. Despite its high melting temperature, the PPS has limited applications due to it having a low glass temperature ($T_{g}$) by flexible sulfide linkages in their own chemical structures and brittleness. In order to overcome these limitations, blending the PPS matrix with other organic/inorganic compounds has been used for preparing the composites [1,2]. 

Since carbon nanotubes (CNTs) with tubular structure of a graphene sheet were firstly reported twenty-five years ago by Iijima et al. [3], The CNTs have been used as a conducting filler to introduce the thermal and electrical conductivity on non-conductive polymers because of their excellent mechanical properties, thermal and electrical properties and having a high aspect ratio [4,5,6,7]. Among various properties of the polymer nanocomposites, thermal conductivity and heat resistance are significant properties applying the polymer nanocomposites to the automotive industry. In order to be used as a thermal resistant material for automobiles, the thermal conductivity should approximately reach 1 to 30 W/mK [8]. The heat resistant material may be a composite material comprising a thermally conductive filler such as ceramic and carbon material. Therefore, several researches have been conducted to improve the thermal properties of various polymers using CNTs as thermal conductive fillers. Kostagiannakopoulou et al. and Ciecierska et al. conducted a study to improve the thermal conductivity from 0.29 W/mK for neat epoxy to 0.36 W/mK for 3 wt % MWCNTs loaded epoxy composites and 0.13 W/mK for neat epoxy to 0.24 for 5 wt % MWCNTs loaded epoxy composites [9,10]. Deng et al. conducted the study to improve thermal conductivity and crystallization properties by introducing carbon based filler such as MWCNTs, and carbon fiber on the PPS matrix [11]. However, the covalent functionalization was widely used for modifying the surface of the MWCNTs because the MWCNTs can be easily aggregated with themselves, which adversely affected the thermal properties of the polymer nanocomposites, due to strong van der Waal’s interactions between individual tubes [12,13]. The hybrid filler system on the polymer matrix has given rise to a new trend for developing higher thermal conductive composite. The hybrid filler system plays an important role in the nature of the interface and the formation of thermally conductive paths in a composite [14]. Therefore, various ceramic fillers have been used in the hybrid filler system on the polymer matrix for enhancing the thermal conductivity and thermal properties of the composites, such as boron nitride (BN), aluminum nitride (AlN), silicon nitride (Si_3_N_4_) [15,16,17], aluminum oxide (Al_2_O_3_), zinc oxide (ZnO), and beryllium oxide (BeO) [18,19,20].

In this study, the AlN was additionally introduced by grafting on the MWCNTs on the PPS matrix. The grafting reaction was chosen to one way to form the hybrid filler system for improving thermal properties, such as thermal conductivity, and thermal resistance by distributing uniformly of the fillers on the polymer matrix [21,22]. The AlN was used as ceramic filler to improve a thermal conductivity. The AlN possesses the low intrinsic thermal resistance, the highest thermal conductivity among the ceramic materials, and good modulus [23,24]. Although the AlN has the high intrinsic thermal conductivity among the ceramic materials, the composites having the AlN particle as the filler exhibit the lower thermal conductivity than the expected, because of their aggregations between the particles [25]. In order to prevent these aggregations, the functional group was introduced by chemical treatments capable of a grafting reaction on the MWCNTs, γ-glycidoxypropyltrimethoxysilane (GPTMS) was used for modifying the surface of the AlN particles.

To the best of our knowledge, the study on the PPS/MWCNTs/AlN composites has not been conducted and reported yet. Therefore, this study aims to describe the effect of the grafting reaction of the AlN particles on the MWCNTs on the distribution on the PPS matrix and thermal properties of the PPS composites.

## 2. Experimental

### 2.1. Materials

The PPS powder used as the polymer matrix of the composite was purchased from Zhejiang NHU Special Materials Co., Ltd., Shaoxing, China. The density of PPS was 1.36 g/cm^3^ at 25 °C. The MWCNTs (CM-130) used in this study were synthesized via a catalytic chemical vapor deposition (CVD) process. They were purchased from Hanwha Nanotech Co., Ltd., Daejeon, Korea. The MWCNTs purchased consists of 15 multiple walls. The outer diameter ranged from 10 to 15 nm, and the aspect ratio was known as $2\times 10^{3}$. The purity and density were >90 wt % and 0.05 g/cm^3^, respectively. Hydrogen peroxide used to functionalize the MWCNTs was purchased from Junsei Chemical Co., Ltd., Tokyo, Japan. The AlN particle having 4 μm mean particle size was purchased from Alfa Aesar Chemical Co., Ltd., Incheon, Korea. Its density was known as 3.26 g/cm^3^ at 25 °C. γ-Glycidoxypropyltrimethoxysilane (GPTMS), and ethanol acetone were purchased from Samchun Pure Chemical Co., Ltd., Pyeongtaek, Korea. Tetrahydrofuran (THF), and acetone used to graft AlN particles on the MWCNTs were purchased from Junsei Chemical Co., Ltd., Tokyo, Japan, and Samchun Pure Chemical Co., Ltd., Pyeongtaek, Korea, respectively.

### 2.2. Covalent Functionalization of the MWCNTs

Dried MWCNTs was added into a 1:1 volume ratio solution of H_2_O_2_/H_2_O. The suspension was continuously stirred for 10 h at 80 °C to introduce the hydroxyl groups on the surface of the MWCNTs. After stirring, the MWCNTs were filtered and cleaned several times with distilled water until the pH of the supernatant became neutral, and the filtered MWCNTs powder was dried at 120 °C in a vacuum oven over-night (covalent functionalized MWCNTs, fMWCNTs) [26].

### 2.3. Surface Modification of the AlN Particles

**Figure 1:**
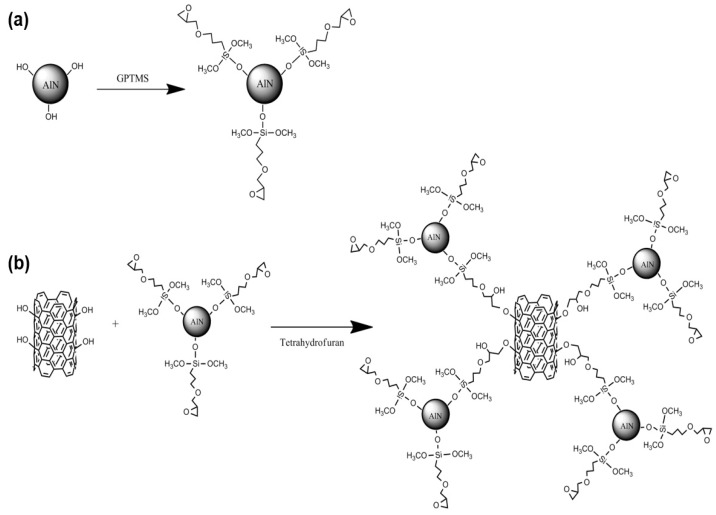
Schemes of the (**a**) silane treatment of AlN and (**b**) grafting reaction of the silane treated AlN on the covalently functionalized MWCNTs (fMWCNTs).

It is necessary to introduce the functional groups which are capable of the rafting reaction with the functional group of the MWCNTs. Therefore, surface modification of AlN particles was performed using a GPTMS as shown in Figure 1a. The AlN particles, and the GPTMS were added into ethanol and stirred continuously at 80 °C for 5 h. The suspension was filtered and washed with distilled water to eliminate non-reacted organosilanes. Then, the filtered AlN powder was dried at 120 °C in a vacuum oven overnight (Silane treated AlN).

### 2.4. The Grafting Reaction of the AlN Particles on the MWCNTs

The grafting reaction of the AlN particles on the MWCNTs was performed as shown in Figure 1b. The fMWCNTs and silane treated AlN were added into THF and stirred continuously at 60 °C for 1 h. The suspension was filtered and washed with acetone to remove the residual solvent. Then, the filtered hybrid filler was dried at 120 °C in a vacuum oven overnight (AlN grafted MWCNTs).

### 2.5. Preparation of the PPS/MWCNTs/AlN Composites

The PPS/MWCNTs/AlN composite was prepared via a melt-blending technique with various contents of AlN particle. The fMWCNTs, AlN, and PPS powders were poured into absolute ethanol. The mixture was sonicated for 5 min in a ultra-sonication bath (SD-250H, 290W, Mujigae Co., Seoul, Korea) and then dried in a vacuum oven at 120 °C until all moisture and solvents evaporated. Then, the PPS/MWCNTs/AlN mixture was melt-blended at 310 °C using a micro-extruder (Wellzoom, Seoul, Korea) and then compressed using a hot press technique with a two-post manual hydraulic press (#2699, Carver Inc., Wabash, IN, USA) at 290 °C under 20 MPa. Finally, the mold was cooled in the air to room temperature until the sample hardened. The sample composition of the PPS/MWCNTs/AlN composite was shown in Table 1.

### 2.6. Characterization

A spectrum One Fourier transform infrared spectrophotometer (Perkin Elmer, Shelton, CA, USA) was used in transmission mode to observe the introduced silane coupling agent on the surface of the AlN particles. The obtained spectra ranged from 400 to 4000 cm^−1^ at a resolution of 4 cm^−1^. XPS measurement was performed using a monochromatic Al Kα X-ray source (15 kV, hν = 1486.6 eV) to confirm the introduction of the silane coupling agents on the surface of AlN particles by observing the Si_2p_ peak and N_1s_ peaks, respectively. Field-Emission Scanning Electron Microscopy (FE-SEM, SU70, Hitachi, Tokyo, Japan), was used to observe the AlN particles grafted on the MWCNTs, and the dispersion differences of the hybrid filler on the PPS matrix depending on the grafting of the AlN on the MWCNTs. Before the FE-SEM characterization, the fractured surface of the composite was sputter coated with a thin layer of platinum. UV–Visible spectrophotometer (UV–Vis, Optizen 2120UV, Mecasys, Daejeon, Korea) was used to confirm that AlN particles was grafted well on the MWCNTs. The suspension was prepared by dispersing 0.2 g powder (grafted or not) in 25 mL ethanol. Then, the suspension was continuously stirred for 30 min at room temperature, and stayed free for 1 day. Field-Emission Transmission Electron Microscopy (FE-TEM, G2 F30 S-TWIN, Tecnai, Eindhoven, Holland) was also used to observe the AlN particles grafted on the MWCNTs. The thermal conductivity of the PPS/MWCNTs/AlN composite was measured using a C-therm TCI thermal conductivity analyzer by the modified transient plane source method (ASTM D7984) at room temperature under normal atmosphere. The measured testing value was averaged five times for each sample. A thermogravimetric analyzer (Q-5000 IR, TA Instruments, Inc., New Castle, DE, USA) was used in order to observe the changes in the thermal resistant property of the PPS/MWCNTs/AlN composites depending on the grafting reaction. The sample was heated from room temperature to 800 °C at a heating rate of 10 °C/min under a nitrogen atmosphere. A differential scanning calorimeter (DSC-Q1000, TA Instruments, Inc., New Castle, DE, USA) was used in order to observe the changes in the crystallinity, crystallization temperature, and melting temperatures depending on grafting. The sample was heated to 310 °C at 10 °C/min and held for 5 min to remove the thermal history within the sample. Then, the sample was cooled to room temperature at 10 °C/min. The second heating run was performed in the same way with the first heating run.

## 3. Results and Discussion

### 3.1. Characterization of the Surface Modification of the AlN Particles

**Figure 2:**
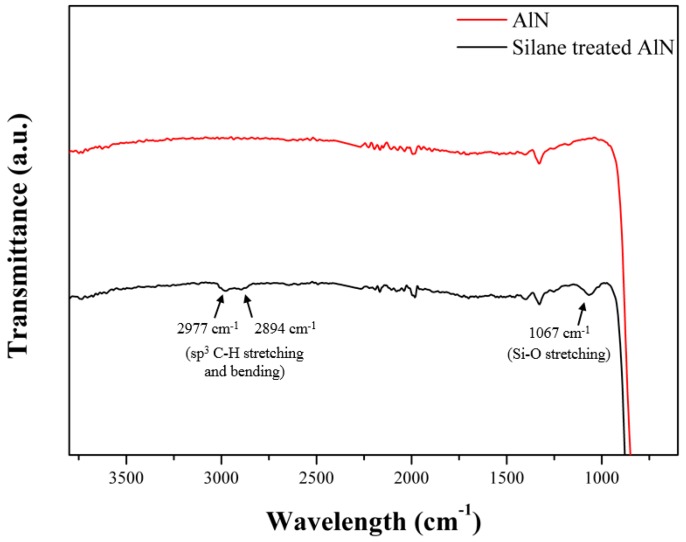
FT-IR spectra of the AlN and silane treated AlN.

As mentioned above, surface modification was performed in order to be able to graft of AlN particles on the MWCNTs. To confirm the GPTMS, silane coupling agent was introduced well on the surface of the AlN particles, the FT-IR spectra of untreated AlN particles and silane treated AlN were compared in Figure 2. In the case of the untreated AlN particles, a broad peak only appeared in the spectrum near 720 cm^−1^ attributed to stretching and bending of the Al–N bond, whereas in the case of the silane treated AlN, additional peaks appeared due to the attached GPTMS. In more detail, two peak appeared at 2977 and 2894 cm^−1^ attributed to stretching and bending of the methylene groups in the attached GPTMS. Moreover, another peak appeared at 1067 cm^−1^ attributed to the Si–O stretching in the GPTMS [27]. As a result, it was confirmed that surface modification was performed well by observing the several peaks of the FT-IR spectrum of the silane treated AlN. 

**Figure 3:**
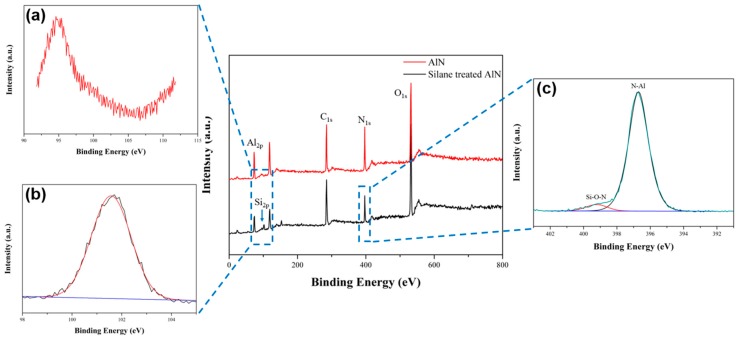
XPS spectra of the AlN and silane treated AlN. (**a**) Si_2p_ peak of the AlN; (**b**) Si_2p_ peak of silane treated AlN; and (**c**) N_1s_ peak of the silane treated AlN.

Additionally, the bonding structure and quantitative analysis of the silane coupling agent introduced on the surface of the AlN particles were performed by XPS analysis simultaneously. The XPS spectrum and elemental composition results were described in Figure 3, and Table 2. In the case of the untreated AlN particles, no peak appeared in the Si_2p_ orbital shown in Figure 3a. On the other hand, in the case of silane treated AlN, the single deconvolution peak appeared at 101.6 eV in the Si_2p_ orbital attributed to the –Si–O–N– bonding shown in Figure 3b. Moreover, two deconvolution peaks appeared at 396.7 and 399.2 eV in the N_1s_ orbital attributed to –N–Al– bonding and –N–O–Si– bonding, respectively (Figure 3c). It was confirmed as a result of the quantitative analysis for the elements that about 4.6 at % of the silane coupling agent was introduced on the surface of the AlN particles [28,29].

### 3.2. Characterization of the Grafting Reaction of AlN Particles on the MWCNTs

**Figure 4:**
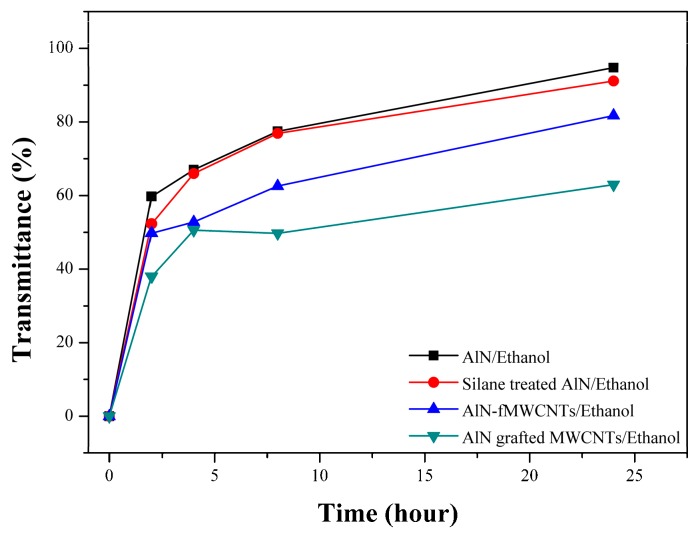
UV–Visible transmittances of the MWCNTs/AlN in ethanol depending on the time.

In order to confirm the AlN particle was actually grafted on the MWCNTs, the change in the transmittance with time in ethanol suspension was observed using UV–Vis as shown in Figure 4. In the case of the silane treated AlN, it was precipitated easily in ethanol similar to the untreated AlN. In the case of the AlN-fMWCNTs, the transmission was slightly reduced due to the improved dispersion of MWCNTs by covalent functionalization in ethanol, but did not decrease much because the grafting reaction did not occur. In the case of the AlN grafted MWCNTs, the transmission was drastically reduced compared with that of the AlN-fMWCNTs. This difference originated from the grafting reaction of the AlN on the MWCNTs, it is clearly demonstrated the AlN particle was grafted well on the MWCNTs.

**Figure 5:**
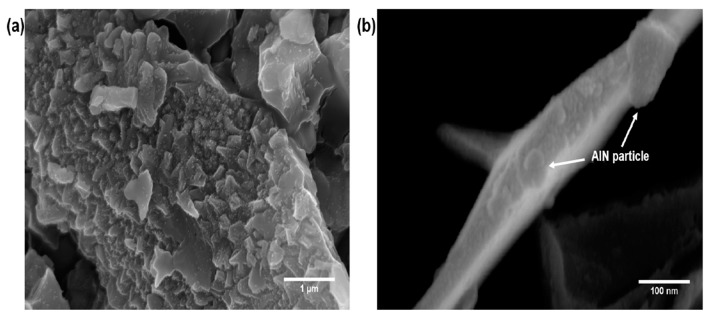
FE-SEM image of (**a**) AlN particles; and (**b**) AlN grafted MWCNTs.

The morphology of the hybrid filler system with the grafting of the AlN particles on the MWCNTs was observed by FE-SEM as shown in Figure 5. Figure 5a,b were the FE-SEM images of the untreated AlN particles and the AlN grafted MWCNTs, respectively. It was confirmed that the grafting reaction of the AlN on the MWCNTs was performed well by observing the attached AlN particles on the surface of the MWCNTs.

**Figure 6:**
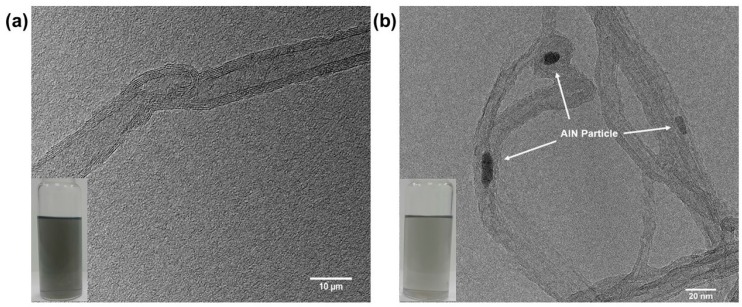
TEM image of (**a**) fMWCNTs; and (**b**) AlN grafted MWCNTs (Inserted images were the MWCNTs dispersion in Ethanol medium).

Additionally, the morphology of the hybrid filler system was observed by TEM as shown in Figure 6. At this time, Figure 6a,b were TEM images of the fMWCNTs and the AlN grafted MWCNTs, respectively. For the morphology of the fMWCNTs, little defects which were formed by introducing the functional groups, were observed on the surface of the MWCNTs. On the other hand, for the morphology of the AlN grafted MWCNTs, the AlN particles having 4 μm particle size were observed on the surface of the MWCNTs. It was further confirmed that the AlN particle was grafted well on the MWCNTs.

### 3.3. Thermal Properties of the PPS/MWCNTs/AlN Composites

**Figure 7:**
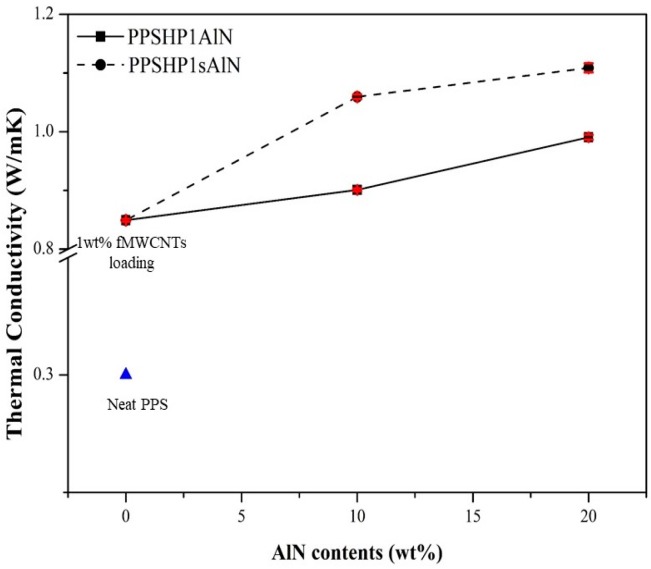
Thermal conductivity of the PPS/MWCNTs/AlN composites.

The thermal conductivity of the polymeric materials emerges by propagating the lattice vibrations generated by interatomic interactions of the phonons within the material [30,31]. However, the phonon propagating through the material is scattered due to the thermal resistance, which comes from phonon-phonon scattering, boundary scattering, and defect or impurity scattering. Therefore, it is most important to minimize the phonon scattering to improve the thermal conductivity [30,32]. To propagate the phonons easily within the material, the heat transfer path is very important. When the filler is introduced in a polymer matrix, its density, shape, particle size, and aspect ratio are the factors affecting the formation of the heat transfer path [30,32,33]. As mentioned in the introduction, the grafting reaction between the fillers was chosen as one way to form the heat transfer path [21,22]. The thermal conductivity of the PPS/MWCNTs/AlN composite depending on the grafting reaction was compared in Figure 7. The thermal conductivity of the PPS/MWCNTs/AlN composite tends to increase in proportion to the AlN contents from the PPSHP1 reported regardless of the grafting on the MWCNTs compared with the neat PPS (0.28 W/mK) and PPSHP1 (0.85 W/mK) [34]. Also, It was originated from AlN grafted MWCNTs was more uniformly dispersed than not grafted on the PPS matrix by observing a higher thermal conductivity value for PPSHP1sAlN than PPSHP1AlN. It was confirmed the AlN grafted MWCNTs acts as a heat transfer path contributed to promote the phonon propagation, which results in a higher thermal conductivity value [35].

**Figure 8:**
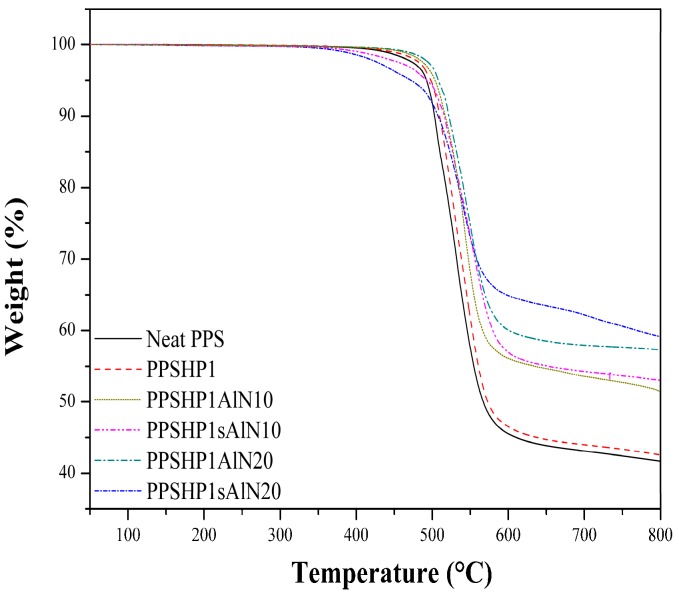
TGA thermogram of the PPS/MWCNTs/AlN composites.

Figure 8 shows the TGA thermogram of the PPS/MWCNTs/AlN composite. The TGA characteristic thermal data for the PPS/MWCNTs/AlN composites were shown in Table 3. The heat-resistance index was the value used to evaluate the thermal resistance of the polymer composites. The heat-resistance index was calculated using Equation (1) with the decomposition temperature ($T_{5}$ and $T_{30}$) from the 5% and 30% weight losses, respectively [36].

(1) $$T_{\mathrm{heat}-\mathrm{resistance}\;\mathrm{index}}=0.49\times \left[T_{5}+0.6\times \left(T_{30}-T_{5}\right)\right]$$

When the fMWCNTs was introduced on the PPS matrix, the heat-resistance index was slightly improved from 252.90 °C for neat PPS to 255.87 °C for PPSHP1. Moreover, it was confirmed that the addition of AlN particles to PPSHP1 highly increased the thermal stability in proportion to the AlN contents due to the intrinsic thermal properties of the AlN regardless of the grafting on the MWCNTs. Interestingly, in case of the PPSHP1sAlN, since the bond between the functional group of the fMWCNTs and the GPTMS introduced on the surface of AlN particles was decomposed between 400 and 500 °C, the heat-resistance index was lower than that of the PPSHP1AlN [37]. 

**Figure 9:**
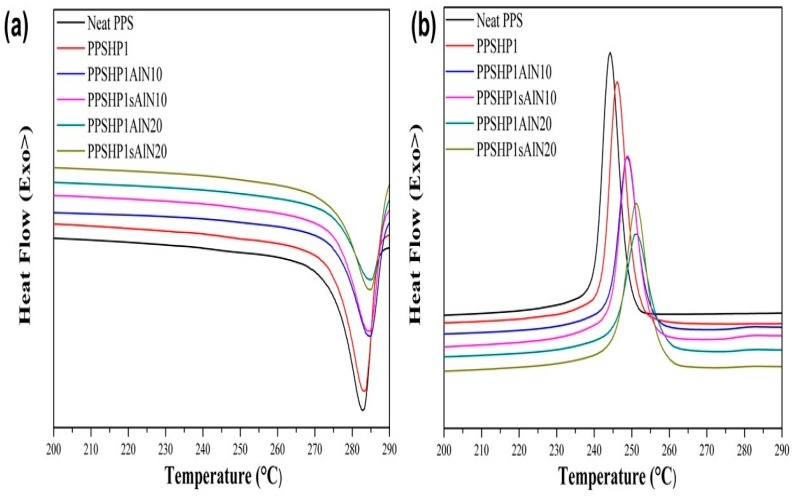
DSC (**a**) melting curve and (**b**) cooling curve for 10°C/min heating rate of the PPS/MWCNTs/AlN composites.

Figure 9 shows the DSC melting and cooling curves of the PPS/MWCNTs/AlN composite. Likewise, the DSC characteristic thermal data of the PPS/MWCNTs/AlN composite was shown in Table 4. When the fMWCNTs was introduced on the PPS matrix, the glass temperature ($T_{g}$), crystallization temperature ($T_{c}$), melting temperature ($T_{m}$), and crystallinity ($\chi_{c}$) were improved due to their outstanding intrinsic thermal properties. The crystallinity was calculated using Equation (2).

(2) $$\chi_{c}=\frac{\Delta H_{f}}{w\times \Delta H_{f}^{0}}$$

where w, $\Delta H_{f}$, and $\Delta H_{f}^{0}$ represent the weight percentage of the PPS, the heat of fusion at the melting point of the PPS/MWCNTs/AlN composites, and the heat of fusion of the crystalline PPS, which was known as 76.5 J/g [38], respectively. Many studies have reported that the MWCNTs, which is a nucleation agent, can facilitate the crystallization by the nucleation effect [11,34,39,40]. The experimental data were consistent with the literatures on the nucleation effect of the MWCNTs. Likewise, the addition of the AlN particles to the PPSHP1 increased the low $T_{g}$ of the neat PPS, which appeared near 85 °C and limited to various applications, was amazingly improved by up to 20 °C. Moreover, $T_{c}$, $T_{m}$, and $\chi_{c}$ were also increased in proportion to the AlN contents due to the intrinsic thermal properties, and nucleation effects of the AlN regardless of the grafting reaction on the MWCNTs. Interestingly, in the case of the PPSHP1sAlN, the $T_{c}$ and $T_{m}$ were not significantly different from the PPSHP1AlN, but the $\chi_{c}$ was further increased by the improvement of dispersion of the AlN particles on the PPS matrix by grafting on the MWCNTs [41].

### 3.4. Morphological Differences of the PPS/MWCNTs/AlN Composites

**Figure 10:**
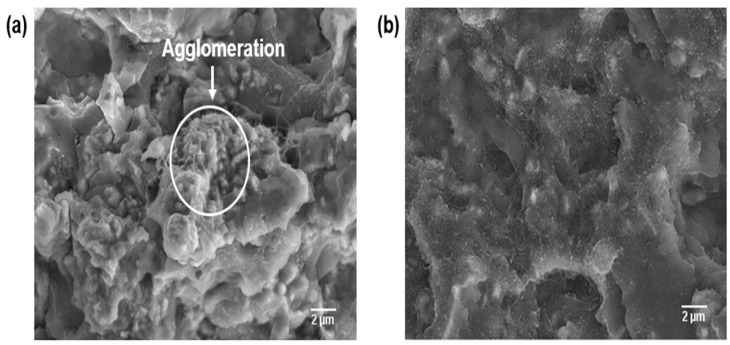
FE-SEM images of the (**a**) PPSHP1AlN20; and (**b**) PPSHP1sAlN20.

The fracture surface was observed by FE-SEM, as shown in Figure 10, to confirm the difference of the dispersion of the hybrid filler on the PPS matrix depending on the grafting of the AlN particle on the MWCNTs. As shown in Figure 10a, the AlN particle was easily aggregated in spherical shape by the interaction between each particle. In the case of the fractured surface of the PPSHP1AlN20, the spherical agglomerated form of the AlN particles was observed [24]. On the other hand, the fractured surface of the PPSHP1sAlN20 was shown in Figure 10b, it was confirmed the homogeneous distribution of the AlN particle was obtained by grafting on the MWCNTs without any agglomerated form between the AlN particles. Therefore, a uniform distribution of the hybrid filler, acting as a heat transfer path allows the phonon to move easily within the material, results in a high thermal conductivity. 

**Figure 11:**
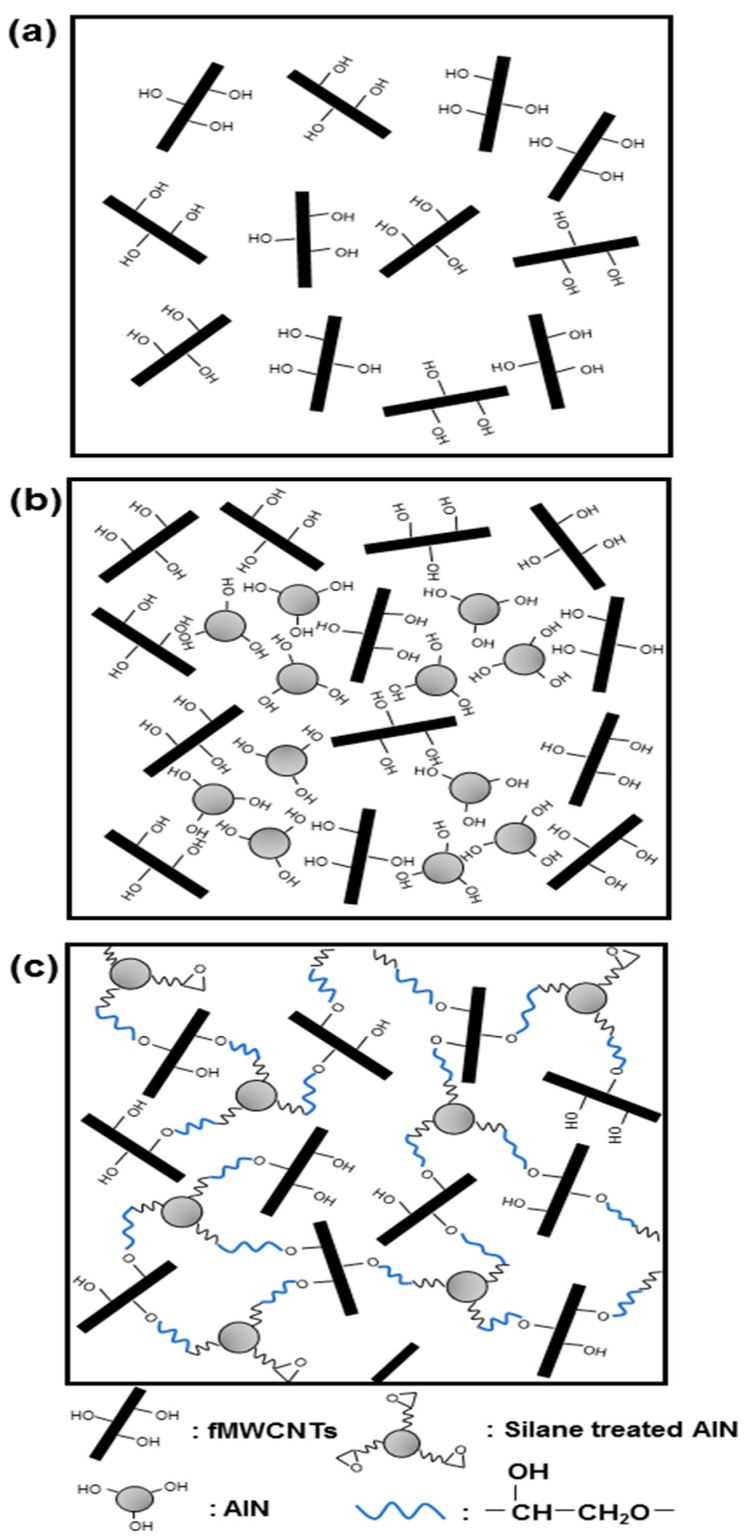
Schemes of the dispersion of the hybrid filler on the PPS matrix.

Based on the results, the actual distribution of the AlN particles and MWCNTs dependent on the grafting reaction was schematized as shown in Figure 11. In the case of the PPSHP1 shown in Figure 11a, the dispersion of the MWCNTs on the PPS matrix was improved by introduced functional groups introduced by covalent functionalization. In the case of the PPSHP1AlN, as shown in Figure 11b, the agglomerated form of the AlN particles, formed by the interaction between each particle, was represented in Figure 10a, while in the case of the PPSHP1sAlN, since the hybrid filler was distributed uniformly on the PPS matrix as seen in the Figure 10b, it can be schematized as shown in Figure 11c.

## 4. Conclusions

In this study, the PPS composite containing the hybrid filler system was prepared via melt-blending techniques. The hybrid filler systems were divided AlN grafted MWCNTs, and not grafted MWCNTs. To be capable of the grafting reaction with the fMWCNTs, the GPTMS was introduced on the surface of the AlN particles, and confirmed by observing new peaks attributed to the GPTMS in FT-IR and new deconvolution peaks of Si_2p_ and N_1s_ orbitals in XPS analysis. The grafting reaction of the AlN particles on the MWCNTs was also confirmed by observing the transmittance change of the hybrid filler system in UV–Vis, and the morphological change of the MWCNTs in FE-SEM and FE-TEM images. The AlN particle was homogeneously distributed on the PPS matrix in the case of the PPSHP1sAlN. This homogeneous distribution of the hybrid filler, acting as a heat transfer path, contributed to improve the thermal conductivity and thermal resistance compared with those of the PPSHP1AlN. Moreover, the glass transition temperature, which is a limitation of the PPS for application in various industries was significantly improved. The melting temperature and the crystallization temperature were also improved compared with those of the PPSHP1AlN. 

## Figures and Tables

**Table 1:** The compositions of the PPS/MWCNTs/AlN composites.

Sample code	fMWCNTs (wt %)	AlN (wt %)	Silane treated AlN (wt %)
PPSHP1	1	-	-
PPSHP1AlN10		10	-
PPSHP1AlN20		20	
PPSHP1sAlN10		-	10
PPSHP1sAlN20			20

**Table 2:** Relative percentages of elemental composition of the AlN and silane treated AlN.

Sample	Elemental composition (at %)				
	Al	N	C	O	Si
AlN	30.8	15.2	28.5	25.5	-
Silane treated AlN	20.7	9.2	35.2	30.3	4.6

**Table 3:** TGA characteristic thermal data of the PPS/MWCNTs/AlN composites.

Sample	Temperature at weight loss (°C)		Heat-resistance index (°C)
	5 wt %	30 wt %	
Neat PPS	492.36	531.97	252.90
PPSHP1	498.12	538.23	255.87
PPSHP1AlN10	503.53	547.58	259.68
PPSHP1AlN20	507.97	558.12	263.65
PPSHP1sAlN10	492.84	555.99	260.06
PPSHP1sAlN20	472.01	558.40	256.68

**Table 4:** DSC characteristic thermal data of the PPS/MWCNTs/AlN composites.

Sample	$T_{g}$ (°C)	$T_{c}$ (°C)	$\Delta H_{c}$ (J/g)	$T_{m}$ (°C)	$\Delta H_{f}$ (J/g)	$\chi_{c}$ (%)	Ref
Neat PPS	89.09	244.28	45.97	282.84	37.06	48.44	[34]
PPSHP1	94.72	246.18	45.22	283.31	40.81	53.89	
PPSHP1AlN10	100.75	248.77	37.69	284.65	34.21	50.25	**-**
PPSHP1AlN20	99.71	251.13	32.40	285.04	30.69	50.78	
PPSHP1sAlN10	102.40	248.97	39.16	284.52	35.56	52.23	
PPSHP1sAlN20	95.29	251.08	39.39	284.79	37.03	61.27	
